# Risk Factors for Diabetic Retinopathy in Latin America (Mexico) and the World: A Systematic Review and Meta-Analysis

**DOI:** 10.3390/jcm12206583

**Published:** 2023-10-18

**Authors:** Oscar Vivanco-Rojas, Sonia López-Letayf, Valentina Londoño-Angarita, Fátima Sofía Magaña-Guerrero, Beatriz Buentello-Volante, Yonathan Garfias

**Affiliations:** 1 Department of Biochemistry, Faculty of Medicine, Universidad Nacional Autónoma de México, Av. Universidad 3000, Mexico City 04510, Mexico; oscarv@bq.unam.mx (O.V.-R.); sonyletayf@comunidad.unam.mx (S.L.-L.); 2 Cell and Tissue Biology, Research Unit, Institute of Ophthalmology, Conde de Valenciana, Chimalpopoca 14, Mexico City 06800, Mexico; valentina.londonan@gmail.com (V.L.-A.); fatima.magana@institutodeoftalmologia.org (F.S.M.-G.); bbuentello@institutodeoftalmologia.org (B.B.-V.)

**Keywords:** diabetic retinopathy, Latin America, meta-analysis, risk factors

## Abstract

Diabetic retinopathy (DR) is one of the main complications of diabetes, and the management of the main control parameters explains only an 11% reduction in the risk of progressing to DR, leaving 89% to be explained by other factors or correlations between the usual factors that are currently unknown. The objective of this systematic review and meta-analysis is to evaluate the similarities and differences between the possible risk factors for developing DR when comparing the world to Latin American populations. The search was performed first for Latin American (LA) populations and a second search for non-Latin American (Non-LA) populations. Using the PRISMA guidelines, five articles were found to be relevant for each of the groups. The patients who had elevated systolic blood pressure (SBP) developed DR more frequently than the patients without retinopathy (Z = 2.1, *p* = 0.03), an effect measured in the population at a global level (GL), behavior that becomes not significant when the LA and non-LA populations are grouped separately; relevant to this is that the diagnosis of hypertension (HBP) grouped globally and stratified does not present a risk factor for DR (Z = 0.79, *p* = 0.42). This indicates that SBP is a risk factor for the world population and that, by separating it into different regions, the omission could cause it not to be considered a possible risk factor. In conclusion, the relationship between the increase in DR associated with the risk factors present in different populations, the limited research conducted in Latin America, and the cultural, social, economic, and genetic differences makes for a complex condition, which reflects the necessity of researching in a more integrated way.

## 1. Introduction

### 1.1. Overview of Diabetes in the World

Diabetes mellitus (DM) is a chronic metabolic disease characterized by elevated blood glucose levels, leading to serious damage [1]. It is estimated that in 2021, approximately 6.7 million adults between 20 and 79 years of age would have died due to complications of DM [2,3]. Among the different complications caused by chronic DM are vascular changes, which are classified into two groups: microvascular and macrovascular complications [4]. Diabetic nephropathy, diabetic neuropathy, and diabetic retinopathy (DR) stand out as the main microvascular complications of DM [5]. DR is recognized as the leading cause of diabetes-associated visual loss among working-age adults and older people worldwide [6,7]. It is currently known that as of 2020, there were 130 million patients with DR, and it has been estimated that the number of patients who will develop DR will approach 160 million by 2045 [8].

### 1.2. Pathology of Diabetic Retinopathy

The pathophysiology involved in DR is characterized by changes such as thickening of the basement membrane of retinal capillaries, increased retinal vascular permeability, tissue ischemia, and the release of proangiogenic molecules such as vascular and endothelial growth factor (VEGF), which have mitogenic properties [9], and autocrine effects on endothelial cells for the survival and viability of retinal cells [10]. Neurodegeneration and changes in glial cells are known to be part of the pathogenesis of DR that occurs in the microvasculature [11]. Some cells exhibit neuroapoptosis in DR, such as retinal ganglion cells (RGCs), a type of neuron that connects the retina to the brain [12]. Macroglia undergo Müller cell proliferation and expression of glial fibrillary acidic proteins, resulting in a reduction in astrocytes, which is associated with increased vascular permeability [13]. DR is a complication characterized by microaneurysms, hemorrhages, exudates, cotton wool spots, venous changes, and neovascularization in the peripheral retina, macula, or both [14]. Recently, therapies have been implemented that are responsible for blocking the activity of VEGF. However, they have not been shown to be effective in the early stages of DR and have different effects [15]. Furthermore, the state of hyperglycemia can cause metabolic changes and the presence of molecules that promote DR, such as sorbitol, which affects retinal capillaries through osmotic changes [16] and increases the hexosamine pathway, causing oxidative stress and consequently increasing vascular permeability and angiogenesis [17]. Additionally, the pathology associated with DR depends on different risk factors, including hyperglycemia, hypertension [18], hyperlipidemia [19], and obesity [20].

### 1.3. Ethnic Differences in the DR

There are reports stating that there are characteristics associated with RD development, such as race and ethnic origin, that modify the prevalence of DR, and not only the global increase or prevalence of DM can do so. For instance, black patients are more susceptible to salt intake, have a reduction in plasma renin activity, and have a higher risk of developing cardiovascular and renal complications than white patients [21]. Additionally, Asians present a higher percentage of body fat, prominent abdominal obesity, a higher content of intramyocellular lipids, and a higher content of liver fat in comparison with Caucasians, regardless of BMI presentation [22]. Other factors, such as glycated hemoglobin (HbA1c) levels, are higher in African-Americans than in white patients [23]. In some cases, the quality of care in the population is an important susceptibility factor in the Hispanic population for the development of DR compared to the white and black populations [24]. Cultural differences and eating habits can be detrimental factors for Latin American groups [25], and some anatomical characteristics, such as the association between wider arterioles and diabetes, were observed mainly in white people; in contrast, the association between wider venules and DM was observed only in Hispanics and Chinese populations [26].

### 1.4. Diabetic Retinopathy in Latin America

Although all ethnic groups are susceptible to common risk factors for DR development, ethnic differences may also influence the early development of this condition. Therefore, research is needed on the predominant factors in each global region that may indicate the early development of DR. Importantly, among all the countries in Latin America, Mexico and Brazil had increased their DM prevalence in less than ten years (7.5% to 13.7% and 6.2% to 7.4%, respectively); thus, both countries are now located in the ten countries with the highest rates of DM worldwide. The main difference between Mexico and Brazil is that the former presents high rates of type 2 DM, while the latter presents high rates of type 1 DM [27]. The Rapid Assessment of Avoidable Blindness (RAAB) study is a global prevention project on vision loss that aims to estimate the prevalence of blindness [28], including DR. The study was performed in 81 countries, including 16 Latin American countries, with a collection of 360 population-based surveys. Although the scientific strategy was well performed, eye fundus examination was performed with direct ophthalmoscopy; therefore, the prevalence of blindness associated with DR may be underestimated, which limits the correct evaluation of DR frequency.

It is also important to consider factors such as the degree of hyperglycemia, hypertension, hypertriglyceridemia, plasma levels of HbA1c, and advanced glycation products (AGE) that each population presents, which may differ according to the area in each region, socioeconomic level, and the quality of health services, making it difficult to establish generalities in DR. However, the impact of reducing these parameters explains only 11% of the reduction in the risk of suffering from RD globally [29]. Thus, 89% of the decreased risk of developing DR is due to other unknown factors, both genetic and environmental, and to other biomolecules not yet related to the development of DR [30]. Furthermore, obtaining attention to risk factors in a single scheme could emphasize the possible relationship between them, according to the different ethnicities in which they are identified. The purpose of this study is to find, through a systematic review and a meta-analysis, the possible DR risk factors that are representative of the Latin American population, contrasting its behavior with the rest of the world, to identify differences or similarities that may be decisive when analyzing the development of DR in Latin America.

## 2. Materials and Methods

For the systematic review, searches were carried out in two databases, PubMed (https://pubmed.ncbi.nlm.nih.gov/ (accessed on 1 May 2023) and Scielo (https://scielo.org/es/ (accessed on 1 May 2023)), between May 2023 and June 2023. The search in Scielo was considered to obtain information on Latin American studies, which may not have been published in journals indexed in PubMed.

### 2.1. Registration

The systematic review was performed following the PRISMA guidelines for systematic reviews and meta-analyses. It is registered in PROSPERO, record ID: CRD42023442428.

### 2.2. LATAM Systematic Search

The search string was (Diabetic Retinopathy [Title]) AND (Country, [All Fields]); filtering by date (11 years), type of article (articles, clinical trials, case reports, and randomized controlled trials), and language (English, Spanish, and Portuguese). The Latin American countries that were included (Antigua and Barbuda, Argentina, Belize, Bolivia, Brazil, Chile, Colombia, Costa Rica, Cuba, Ecuador, El Salvador, Guatemala, Haiti, Honduras, Jamaica, Martinique, Mexico, Nicaragua, Puerto Rico, Panama, Paraguay, Peru, the Dominican Republic, Trinidad and Tobago, Saint Barthelemy, Saint Lucia, Saint Kitts and Nevis, Suriname, and Venezuela). The same search was performed in Scielo, with the difference that countries can be filtered and do not need to be added to the search string.

### 2.3. Non-LATAM Systematic Search

The search string was (Diabetic Retinopathy [Title]) filtering by date (11 years), type of article (articles, clinical trials, case reports, and randomized controlled trials), and language (English, Spanish, and Portuguese). Searches were performed by two independent observers (OVR and SLL).

### 2.4. Inclusion and Removal Criteria

For the inclusion of articles, those that contained tables with demographic and clinical information were included, and articles with nonhuman information were excluded, as well as duplicates and articles without relevant information for the present study. The articles were selected in the first instance only to admit patients with a diagnosis of type 2 diabetes. Based on the grouping, DR vs. no DR exclusively and clinical parameters were extracted: diagnosis of hypertension (HBP), dyslipidemia (DLP), smokers (SMK), values of systolic blood pressure (SBP), diastolic blood pressure (DBP), high-density lipids (HDL), low-density lipids (LDL), and glycated hemoglobin (HB1AC).

### 2.5. Quality Assessment

Bias assessment of the primary outcomes was assessed using the Cochrane Risk of Bias (ROB) 2.0 tool. It evaluates five quality characteristics of RCTs: randomization process, deviations from intended interventions, missing outcome data, measurement outcome data, and selection of the reported outcome [31]. Studies were classified as low risk if they met all domains with a low risk of bias, high risk if they met any domain with a high risk of bias, and as having some concerns if they did not fit into any of the categories. Two independent reviewers (OVR, SLL) assessed the included studies, and a third reviewer (YG) resolved any discrepancies.

### 2.6. Data Extraction

Data from each article were extracted in duplicate by two independent observers (OVR and VLA), discrepancies were resolved by a third observer (YG) or by consensus, and data per variable were written as follows: Diagnosis of HBP, DLP, and SMK as dichotomous variables present or absent. DBP and SBP are continuous variables measured in mmHg as the mean with standard deviation (SD), HDL/LDL are continuous variables measured in mmol/L as the mean with SD, and Hb1Ac is a continuous variable measured as a percentage in the mean with SD.

### 2.7. Statistical Analysis

Data were analyzed by two programs: R (The R Foundation for Statistical Computing, Vienna, Austria) and Jamovi (The Jamovi Project, Sydney, Australia). For dichotomous data analysis (HBP, DLP, and SMK), analyses were performed using the log-odds ratio as the outcome measure. For continuous data (SBP, DBP, HDL, LDL, and HB1AC), this was performed using the standardized mean difference as the outcome measure.

### 2.8. Meta-Analysis

For the analysis of the populations, they were organized into 3 groups: global level (GL), which includes both populations; a group focused on Latin America (LA); and the last, excluding LA (non-LA), using a random effects model. The data were adjusted considering that there was a difference in some risk factors in each population with a value of *p* < 0.05 (Z, *p* < 0.05). The amount of heterogeneity (tau^2^ = 0) was estimated using the maximum likelihood estimator (MLE). In addition to the tau^2^ > 0 estimate, the Q test for heterogeneity and the I^2^ statistic are reported. To verify the asymmetry of the funnel plot, the rank correlation test (Begg and Mazumdar Rank Correlation, *p* > 0.05) and the regression test (Egger Regression, *p* > 0.1) were used, using the standard error of the observed results. Analyses were performed using R study 1.3.1093 software.

## 3. Results

**Figure 1:**
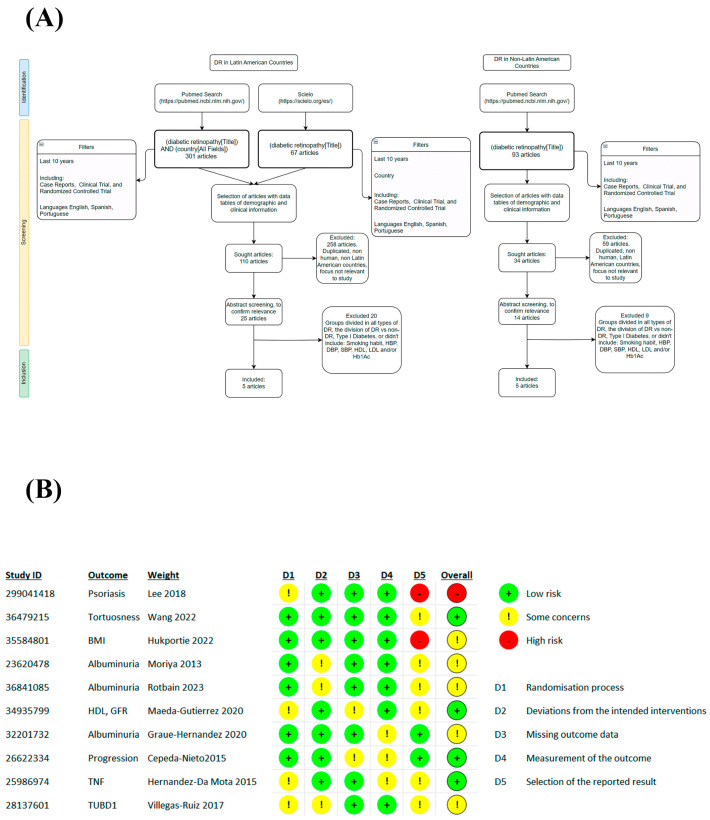
Flow diagram and BIAS. Based on PRISMA guidelines (**A**) and quality evaluation using the ROB 2 tool (**B**) [32,33,34,35,36,37,38,39,40,41].

To obtain the final articles for the meta-analysis, we managed them according to PRISMA guidelines, as shown in Figure 1.

The results of the search and data collection are shown in the following link: https://www.dropbox.com/scl/fi/ake7gnij14pbis94ayyta/Base-de-datos-LA-vs-Non-LA.xlsx?rlkey=9yia3wdixkoz306bmwwsvq2sv&dl=0 (accessed on 18 September 2023).

In the systematic search, a total of 10 studies were selected at the global level (GL) and stratified in the non-Latin American population (Non-LA) with 5 studies, of which there are countries such as the Republic of Korea (KR), China (CHN), Japan (JPN), and another that handles populations from different continents labeled international (INT), while for the Latin American population (LA), 5 studies were obtained, of which only the Mexican population (MXN) was found. From these studies, a total GL general population of 1,817,539 patients was derived, of which 1,798,317 patients did not present retinopathy and 19,222 patients presented some type of retinopathy. Stratification in the non-LA population was 1,814,933 total patients, 1,797,053 people without DR, and 178,860 with DR. For the LA population, there were 1826 patients, of whom 1264 did not present DR and 562 presented some type of DR. The characteristics of the studies are summarized in Table 1. The search revealed that the articles that represented Latin America (LA) only focused on the Mexican population, from which studies were found focused on the analysis of the risk factors involved in the development of DR and clinical studies, finding a positive association with the levels of HDL, creatinine, glomerular filtration ratio, cholesterol [32], SBP, HbA1c, and albuminuria [33], proinflammatory cytokines such as TNF-a and IL-6 [34], mRNA such as TUBD1 [35], and others ruled out the association that could exist between DR and some integrin a2 polymorphisms [36]. In the studies in non-LA, the approach was differentiated between imaging trials analyzing values of venule caliber, arterial tortuosity, insulin dose, and duration of DM [37], while others associated it with clinical characteristics such as albuminuria [38,39], another focused on morphometric values such as body mass index and waist circumference [40], and another was related to conditions that may be important factors in DR such as psoriasis [41].

### 3.1. DBP as a Possible Risk Factor for DR in a Non-Latin American Population

**Figure 2:**
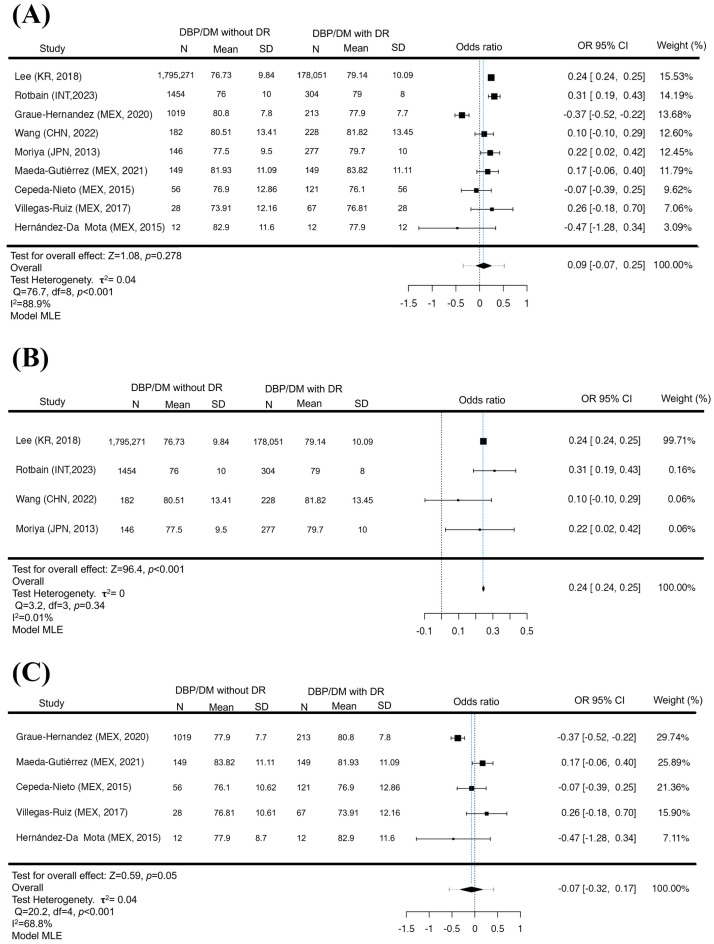
DBP in DR. Forest plot of DBP between diabetic patients with and without DR. The presence of elevation in DBP is not a risk factor for presenting DR (*p* = 0.34) (**A**). The exclusion of Latin America with elevated DBP is a factor in the appearance of DR (*p* < 0.001) (**B**). In contrast, the Latin American analysis did not show a significant difference between DBP pressures and the presence of DR (*p* = 0.54) (**C**). Maximum Likelihood Model (MLE) [32,33,34,35,36,37,38,39,41].

In the HBP meta-analysis, no significant difference was found in the groups analyzed for GL (*p* = 0.42), non-LA (*p* = 0.81), and LA (*p* = 0.06), which would indicate a possible risk factor for suffering DR (We complete this to Figure S1). Although the variance between the studies is increased in those that represent LA due to the reduced population compared to the non-LA, which maintains a lower variance in HBP, this increased variance was found in several of the studies in LA. A total of N = 9 studies were included in the DBP meta-analysis. The observed standardized mean differences were positive estimates (67%), with an estimated mean based on the random effects model of 0.09 (95% CI −0.075 to 0.249). The average result did not differ significantly from zero (*p* = 0.34), which indicates that there is no relationship between the increase in DBP and the presence of DR at a global level (Figure 2A). While in the non-LA analysis (N = 4), most estimates were positive (100%), the estimated average standardized mean difference based on the random effects model was 0.2443 (95% CI 0.2394 to 0.2493). Therefore, the average result differed significantly from zero (*p* < 0.001). This suggests that the increase in DBP is a possible risk factor for the development of DR if the Latin American population is excluded (Figure 2B); otherwise, when stratifying LA (N = 5), most of the estimates were negative (60%). The mean difference based on the random effects model was −0.0739 (95% CI −0.3177 to 0.1698), and the mean result did not differ significantly from zero (*p* = 0.54), showing that increased DBP is not a factor for the development of DR in the Latin American population (Figure 2C). It is noteworthy that the analysis of DBP between the different groups (GL, non-LA, LA) was variable, between positive and negative values of the confidence intervals, which indicates that the studies became contradictory, generating a dispersion of data at the time of analyzing the DBP in the GL and LA populations; otherwise, the non-LA population presented a positive behavior with a significant difference. Neither the rank correlation nor the regression test indicated asymmetry in the funnel plot in the analyses (*p* = 0.35/*p* = 0.29, *p* = 1.0/*p* = 0.91, *p* = 0.75/*p* = 0.69, respectively).

### 3.2. Increased SBP as a Risk Factor for DR for GL

**Figure 3:**
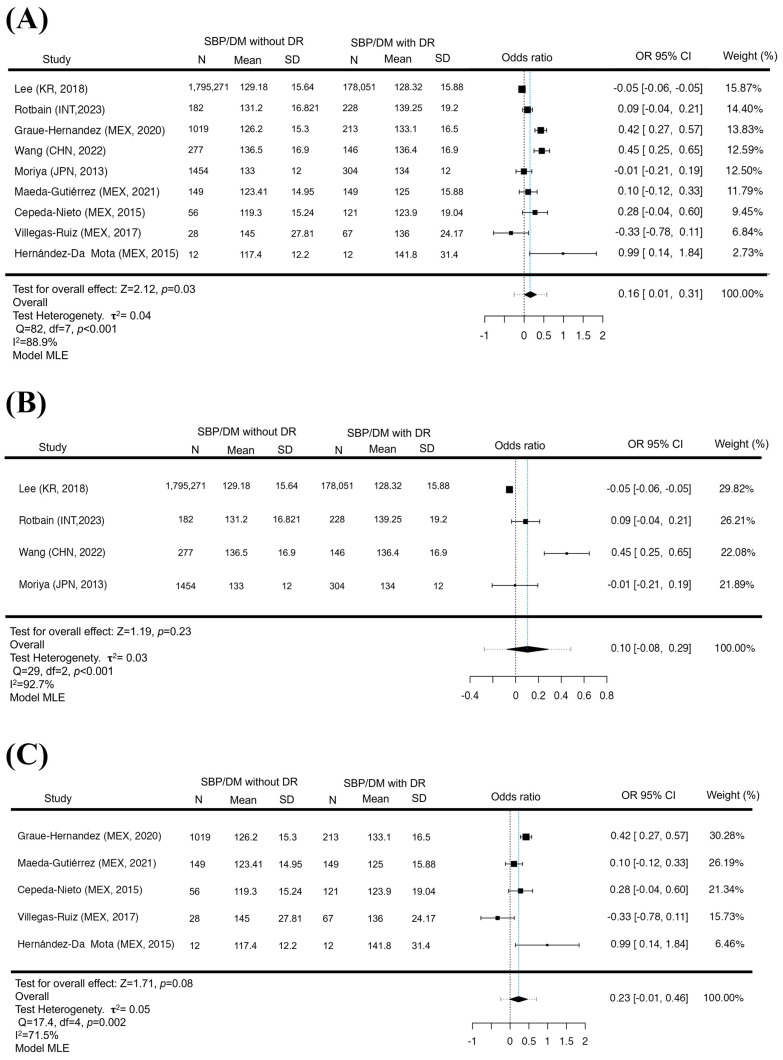
SBP increased in GL but not in LA and non-LA. A forest diagram of SBP between patients with and without DR. The presence of elevation in SBP is a risk factor for the development of DR (*p* = 0.03) (**A**). With the exclusion of Latin America, elevated SBP was not a risk factor for the appearance of DR (*p* = 0.23) (**B**). As in the Latin American population, there was no significant difference between SBP pressures and the presence of DR (*p* = 0.08) (**C**). Maximum Likelihood Model (MLE) [32,33,34,35,36,37,38,39,41].

The analysis of SBP for GL found that of a total of N = 9 studies, most of the estimates were positive (67%), similar to what was found in the analysis of the DBP (67%), but with the difference that the estimated mean standardized means based on the random effects model was 0.1607 (95% CI 0.0069 to 0.3145). Therefore, the average result differed significantly from zero (*p* = 0.030), suggesting that elevated SBP may be a risk factor for the development of DR for GL. However, when removing the Latin American population (N = 4), it was found that the standardized mean differences were mostly negative estimates (50%). The estimated mean difference based on the random effects model was 0.1045 (95% CI: −0.0774 to 0.2864), and there was no significant difference that would link increased SBP as a risk factor in the non-LA population. (*p* = 0.23), in this context, although the Latin American population (N = 5) presented positive estimates (80%). The estimated mean difference based on the random effects model was 0.2257 (95% CI −0.0134 to 0.4648). Elevated systolic blood pressure did not represent a risk factor for the development of DR (*p* = 0.08). In the SBP, the analysis of each group allowed us to observe that in all of them there is a tendency to be a determining factor for developing DR. However, only a significant difference was statistically found for GL, which indicates that to analyze the SBP, it is necessary to consider the world population (Figure 3). However, the LA population has values that could be considered a risk factor in the Latin American population, but one must be discreet due to the values of confidence intervals that become negative and positive. In the rank correlation and regression tests, GL and LA did not indicate asymmetry in the funnel plot (*p* = 1.0, *p* = 0.2, *p* = 0.81, *p* = 0.65, respectively). For the non-LA population, the regression test indicated asymmetry of the funnel plot (*p* < 0.0001) but not the rank correlation test (*p* = 0.75).

For the analysis of HBA1C, HDL, and LDL (We complete this to Figure S2) and SMK and DLP (We complete this to Figure S3), 5 studies were added to the meta-analysis, and they were not stratified due to a lack of clinical parameters in the studies, so they were only analyzed globally. In this sense, some relationships already reported in systematic reviews and meta-analyses were found, in which the increase in the percentage of glycated hemoglobin is a factor for the development of DR (*p* = 0.002). SMK and HDL (We complete this to Figure S2) were also found to present a risk factor for DR (*p* = 0.01, *p* < 0.001, respectively), although the weight influence of the [41] study was approximately above 90%, and even excluding it from the analysis, it maintained its significance (*p* < 0.05). For LDL and DLP (*p* = 0.83 and *p* = 0.77, respectively), no significant difference was found to indicate possible risk factors for DR.

## 4. Discussion

Our study showed that the diagnosis of HBP is not a risk factor for presenting DR in the world population; furthermore, this statement remains even when the LA population is analyzed independently. However, for SBP and DBP measurements, the picture changes since high levels of SBP are considered a risk factor for DR for the world population, as is high DBP, with the difference that it only applies when it excludes LA. However, the weight of the studies presents a skewed distribution, which makes it difficult to assert that both factors have a similar behavior. For the other parameters analyzed, we found a lack of consensus in the way they are reported, so they could only be analyzed for the world population, which showed that smoking and high HDL could also be considered risk factors only for the world population.

The main limitation of the search for risk factors associated with the development of DR presents complications in terms of systematic reviews focused on a specific population. Due to the lack of studies that provide specific or similar information, in some cases the small population N and insufficient data on clinical features, among others, make it difficult to analyze possible risk factors that could be involved in the diagnosis or prevention of DR. Furthermore, the lack of specific research in each Latin American country makes it difficult to correlate and compare different ethnic groups; the low participation of governmental and nongovernmental institutions causes a delay in the analysis and publication of research on DR [42] that would be of vital importance for the treatment or prevention of this condition.

In our study, we found that the diagnosis of HBP was not considered a risk factor in the population. Although it has been reported that the control of hypertension works as a favorable prognosis to avoid the development of DR, the treatment of HBP is based on the values measured based on systolic pressure. This coincides with our findings where elevated SBP correlates with the appearance of DR and not necessarily with changes in DBP or even with the diagnosis of HBP [43]. Similarly, other studies have found that elevated SBP is a risk factor for the progression or worsening of already-resolved DR [44]. It has even been proposed as a factor that promotes the appearance of proliferative DR and the different complications after the appearance of DR. It has been proposed that the elevation of SBP in association with the state of hyperglycemia results in a deregulation of different metabolic pathways. Affecting the nonenzymatic glycation process in the formation of Amadori products, which are initially the basis for the generation and accumulation of advanced glycation products (AGEs) that play an important pathophysiological role in DR, damage to the blood vessels of the retina is caused by the accumulation of AGEs ions within the cells and in the extracellular matrix, which leads to hardening of the blood vessels, which does not allow them to vasodilate and cause them to rupture or become blocked and cause a reduction in blood flow. Additionally, AGEs cause mitochondrial deterioration, and both events cause retinal hypoxia [45].

At the same time, HBP causes damage to the microvasculature, constricting vasoconstriction and modifying blood flow. These types of changes have a particular impact on different ethnicities, since in white populations with diabetes, they present an increase in the caliber of the arterioles [46], while in Hispanics and Chinese individuals, the changes are in the venular caliber [26], which suggests that the change in SBP and DBP should pay greater attention to the population studied without detracting from the global standards of measurement and care of HBP. Increased blood flow means an increase in laminar flow pressure in the blood vessels, which maintains VEGF expression and does not allow new blood vessels to resolve when the hypoxic event occurs, leading to neovascularization. Some studies support this idea by confirming that endothelial dysfunction caused by high blood pressure can lead to increased VEGF expression, knowing that the incidence of hypertension can vary between African Americans, Hispanics, and whites [47]. It is even more interesting that in Caribbean populations such as Cuba [48], where a mixture of ethnicities has reported that hypertension rates are similar among whites (43%), and blacks (46%), supporting the idea that a better ethnic approach is necessary in the measurement of risk factors commonly analyzed worldwide.

Studies have made it clear that controlling HBP is not enough, but controlling small variations in daily peaks in blood pressure and pulse rate can also lead to reducing the onset and progression of DR [49]. It is important to emphasize that even HBP based on SBP and DBP values should be monitored periodically since it has been reported that they can be independent risk factors for the development of DR [50], coupled with the control of glucose levels, which in excess cause the formation of AGEs that have been shown to cause apoptosis of retinal pericytes and an upregulation of VEGF. Therefore, the evaluation of Hba1c levels works as a test for diagnosing diabetes. Although elevated levels of Hba1c show a direct relationship to the development or complication of DR, this is largely due to the formation of AGEs, which are the last phase of its formation and have irreversible effects on proteins during the development of DR.

This increases interest in continuing to measure and establish the importance of HBA1C levels in patients and the complications they trigger in metabolism at the arterial level. This is more relevant when analyzing it in different populations, considering that elevated levels of HBA1C are higher in Hispanic and Asian people than in Caucasians [23]. Although much of the discussion and evaluation of the factors resulting from the review and meta-analysis lies in publications that are involved at a global level, it is difficult to integrate more detailed information in Latin America.

It has recently been integrated into the RAAB surveys for DR; however, the parameters used to diagnose DR are insufficient according to diagnostic standards. Additionally, in these RAAB surveys, we identified that the majority of the population results are from Mexico, with more than 18,000 analyzed subjects, in contrast to the rest of Latin American countries, which range from 2000 to 8000 subjects per country. Moreover, in RAAB surveys, Mexico is considered to be part of the Caribbean and North America instead of Latin America. The publications of results in scientific journals derived from these surveys are six in total in Latin America, with Mexico being the most publishing country with three of them and the other three being from other countries in Latin America, which suggests that there is a lack of scientific communication of the collected data from these surveys in our region. Furthermore, there is an absence of scientific literature related to DR risk factors in the Mexican population. Most of the studies have been performed elsewhere; for instance, Teo et al. reported a prevalence of 33% of DR in the Mexican population [8], while in a Mexican-American population studied in the USA, a 48% prevalence of DR has been reported [51]. Accordingly, ethnic, cultural, and regional differences must be considered when analyzing DR risk factors. In this regard, a study conducted in Los Angeles, California, found that in a Latin American population, risk factors such as male sex, insulin treatment, hypertension, and longer duration of DM were associated with the development of DR. Latino men had a fourfold increased risk of developing DR compared to Latino women. Additionally, Latino men with systemic hypertension had a 1.6-fold increased risk of developing DR [52]. Being a region with a high prevalence of diabetes among all its ethnic groups, countries such as Brazil [53] mainly study type 1 diabetes or types 1 and 2 diabetes, which are analyzed indistinguishably. However, in Mexico, the prevalence in most of the population is type 2 [54]. This is because in the Mexican population, there is also a high rate of HBP [55], which makes it more attractive to analyze not only the diagnosis of HBP but also the monitoring of SBP and DBP parameters, as well as the variation of these parameters daily. Even treatment against HBP could show their relevance in attempts to reduce the development or progression of DR.

## 5. Conclusions

Therefore, early factors such as SBP, HB1AC, and HDL should be considered crucial factors associated with the different vasculogenic complications of DR. Paying attention to other factors that can be measured routinely could have a greater impact and help take corrective measures and prevent them. reduces the appearance or complication of DR. The relationship between the increase in DR associated with the risk factors present in different populations, the limited research conducted in Latin America, and the cultural, social, economic, and genetic differences makes for a complex condition, which reflects the necessity of researching in a more integrated way. However, most importantly, it is necessary to increase awareness about diabetes and its complications in Latin American countries and promote a system to record clinical data that can be used for future analysis to help create a better understanding of risk factors and biomarkers present in these populations.

## 6. Future Research

The findings suggest the importance of implementing studies focused on the Latin American population, specifically on the factors associated with the development of DR. The creation of open access databases and/or repositories that report and update these data and that allow the creation of efficient machine learning models that apply to Latin America, which allow preventing the development of DR.

## 7. Limitations

This work is not without limitations. Most of the included studies lack a representative population from Latin America, which introduces a bias exclusive to Mexico. Although the statistical analysis suggested the possibility of a small publication bias, the heterogeneity of the investigations must be considered, so the results of this meta-analysis should be interpreted with caution. While some risk factors were present in non-LA and LA populations, allowing their separation into groups, some other values, such as smoking and dyslipidemia (HDL, LDL), were only analyzed globally due to a lack of research. In others, such as glycated hemoglobin, it has been studied routinely, which omits its publication and reduces the analysis between different populations. The absence of risk factors and possible interactions would allow us to evaluate the development of DR.

## Figures and Tables

**Table 1:** Summary of the selected articles.

Author	Country	Risk Factor or Biomarker Studied	Total N	Relevance for DR
Maeda-Gutiérrez V et al. [32]	Mexico	Analisys of risk factors using machine learning to select features and create predictive models for DR	298	Feature selection for the prediction of high-risk patients using common measurements of diabetes, is a quick and cheap way of creating accessible diagnostic aids, specially when there is no access to specialized equipment
Graue-Hernandez EO et al. [33]	Mexico	Several metabolic risk factor were analized in realtionship to recent DR diagnosis and Diabetes duration	1232	The population analyzed was Mexican, and it demonstrated the association of DR with diabetes in patients recently diagnosed with Diabetes type 2, meaning that for this population screening for DR at the moment of diagnosis is necessary. It also noted that for this sample lack of glucose control and high SBP were also associated risk factors for DR.
Cepeda-Nieto AC et al. [36]	Mexico	Integrin α2 gene polymorhisms	177	The polymorphism known as 807T and 807C associated with restriction zones BglII/NdeI has been associated in Japanese and Caucasian population as a risk factor for DR but are not present in the Mexican population analyzed.
Hernández-Da Mota SE et al. [34]	Mexico	Pro-inflammatory citokines, TNF-α, IL-1β, and IL-6, and pro-inflammatory biomarkers, CRP, and globular sedimentation rate.	24	The inflammatory process is considered part of the pathogenesis of DR, in the population studied TNF-α was higher in patients with DR.
Villegas-Ruiz V et al. [35]	Mexico	TUBD1 isoform a and b	95	In the analysis made by the authors, they discovered that this two isoforms were elevated in patients with DR, and that the isoforms were regulated by HIF-1 a molecule also involved in the regulation of VEGF
Lee JH et al. [41]	Republic Korea	Psoriasis risk in patients with DR and ESRD	1,973,322	Psoriasis was prevalent among patients with DR and ESRD, this is relevant because psoriasis being an inflammatory disease, confirms that there is presence of an inflammatory process in diabetic patients, also that the effects of angiogenesis are not limited to retina and might be a systemic issue for this patients.
Wang M et al. [37]	China	Retinal geometry to assess DR	410	In this study during the analysis of their model to assess DR, found like other studies that one of the most important risk factors for DR is duration of diabetes
Hukportie DN et al. [40]	International	Waist circunference and BMI	6217	This study didn’t find correlation between waist circumference and BMI and DR
Moriya T et al. [39]	Japon	Macroalbuminuria risk in relation with DR	423	DR might reflect on the microvascular state of the kidney, leading to lower GFR and in consequence macroalbuminuria.
Rotbain Curovic V et al. [38]	International	Albuminuria	1758	Presence of DR, may increase the risk of presenting macroalbuminuria.

DR, Diabetic Retinopathy; SBP, Systolic Blood Pressure; ESRD, End-Stage Renal Disease; BMI, Body Mass Index; GFR, Glomerular Filtration Rate.

## Data Availability

https://www.dropbox.com/scl/fi/ake7gnij14pbis94ayyta/Base-de-datos-LA-vs-Non-LA.xlsx?rlkey=9yia3wdixkoz306bmwwsvq2sv&dl=0 (accessed on 18 September 2023).

## Supplementary Materials

The following supporting information can be downloaded at: https://www.mdpi.com/article/10.3390/jcm12206583/s1, Figure S1: Forest plot of HBP between diabetic patients with and without DR. The diagnosis of HBP is not a risk factor for DR (*p* = 0.79) (A). In the same way, omitting Latin America, HBP is not a factor for developing DR (*p* = 0.23) (B). Although the Latin American analysis did not show a significant difference between suffering from hypertension and the presence of RD, the differential values of p (0.06) are somewhat borderline compared to the global level, excluding LA (C). Maximum Likelihood Model (MLE); Figure S2: HBA1C, HDL, and LDL forest diagrams with and without DR. Glycated hemoglobin is a risk factor for DR, as has already been reported in other global studies (*p* = 0.002) (A). While HDL levels are a possible risk factor for DR (*p* < 0.001) (B). While LDL levels as reported above did not present a factor to develop DR (*p* = 0.83), globally (C). Maximum Likelihood Model (MLE); Figure S3: DLP and SMK forest plots with and without RD. The diagnosis of dyslipidemia is not a risk factor for DR, which is contradictory, as has already been reported in other studies globally (*p* = 0.77) (A). While smoking is a possible risk factor globally for DR (*p* < 0.01) (B). Maximum Likelihood Model (MLE).
