# Supplementary material for: Risk Factors for Diabetic Retinopathy in Latin America (Mexico) and the World: A Systematic Review and Meta-Analysis
Source: J Clin Med. 2023 Oct 18;12(20):6583. doi: 10.3390/jcm12206583 (PMC10607496; doi:10.3390/jcm12206583)
Supplement: Supplementary file 1 [file jcm-12-06583-s001.zip › jcm-2647898-supplementary.pdf]

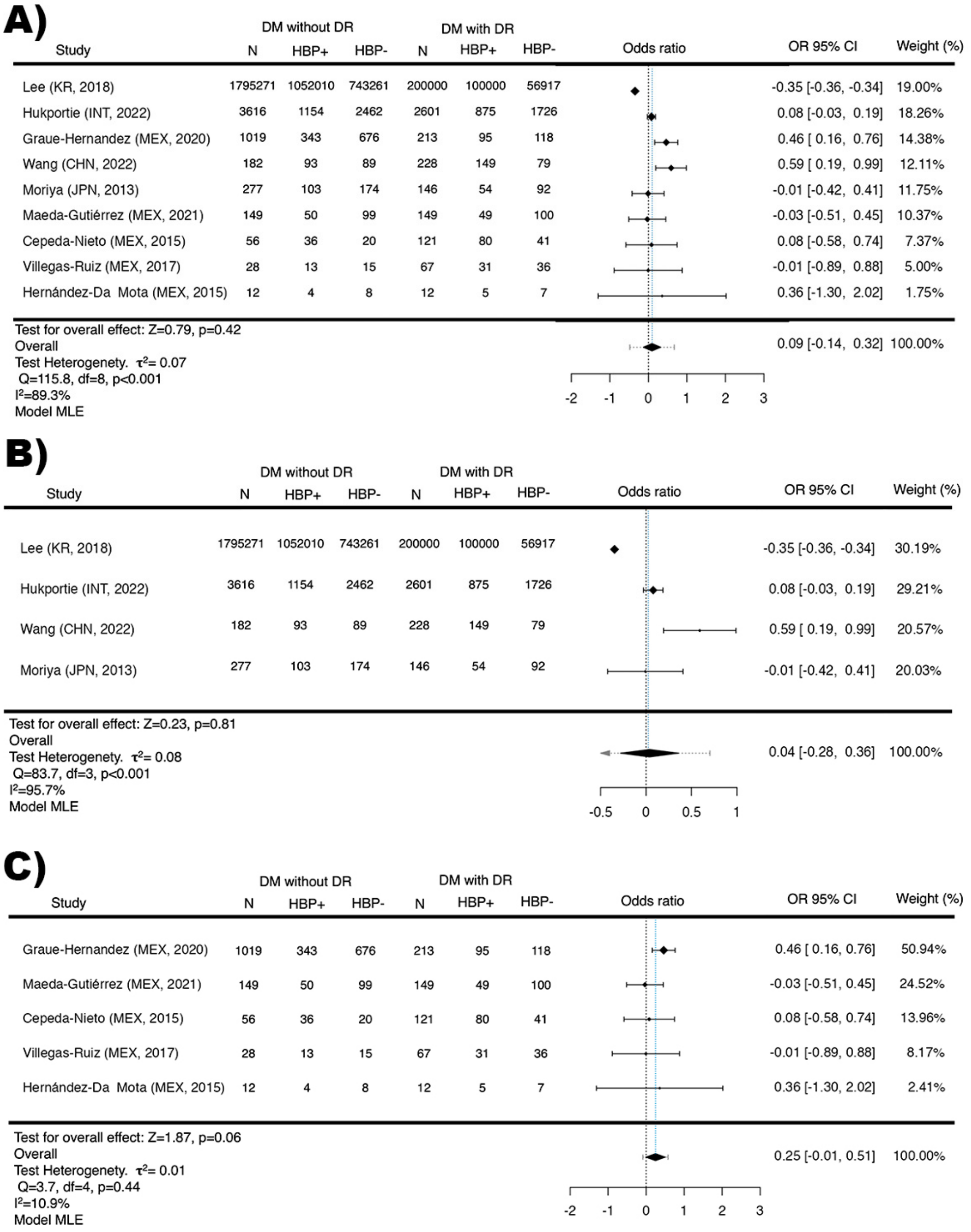

Supplementary Figure S1. Forest plot of HBP between diabetic patients with and without DR. The diagnosis of HBP is not a risk factor for DR (p=0.79) (A). In the same way, omitting Latin America, HBP is not a factor for developing DR (p=0.23) (B). Although the Latin American analysis did not show a significant difference between suffering from hypertension and the presence of RD, the differential values of p (0.06) are somewhat borderline compared to the global level, excluding LA (C). Maximum Likelihood Model (MLE).

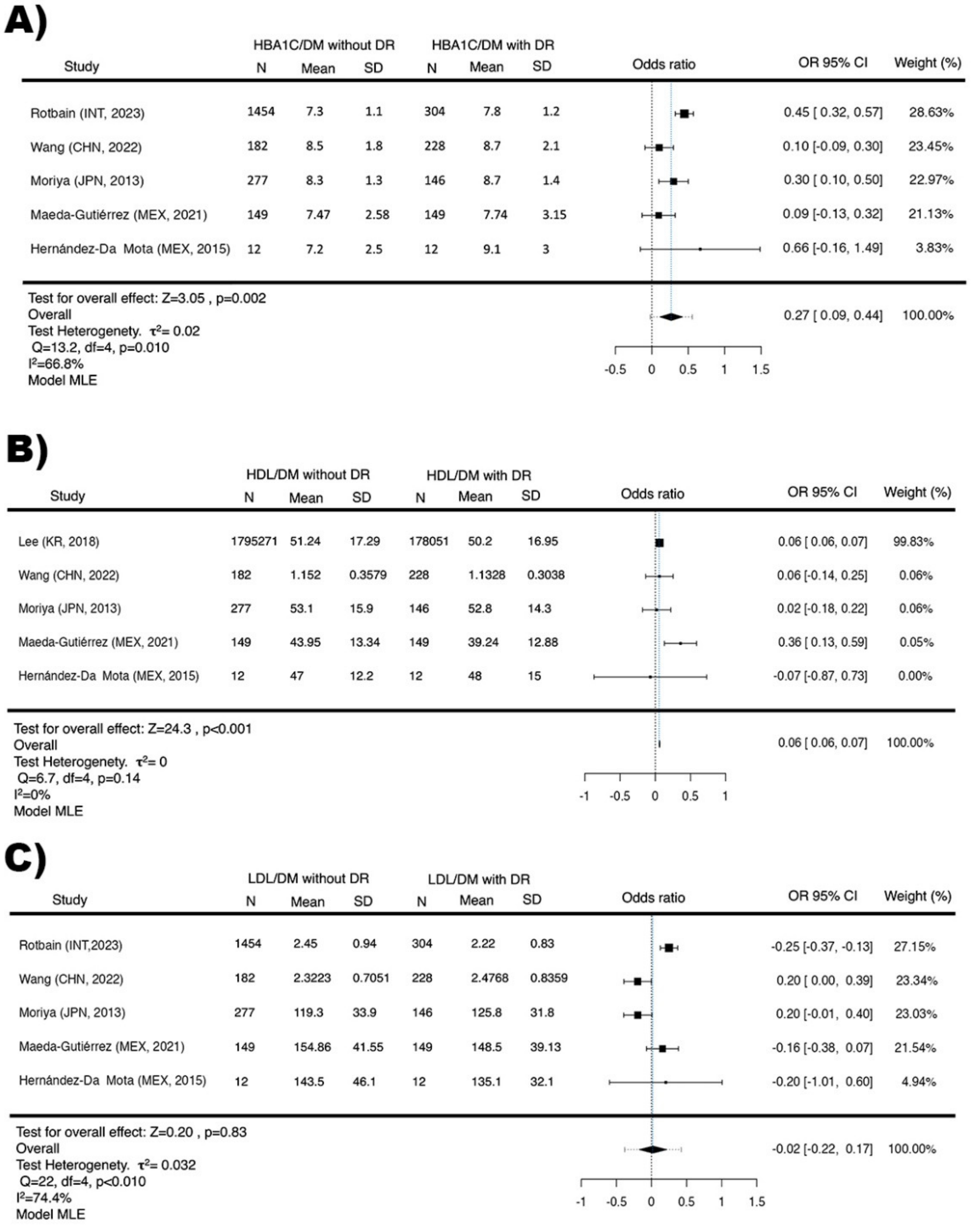

Supplementary Figure S2. HBA1C, HDL, and LDL forest diagrams with and without DR. Glycated hemoglobin is a risk factor for DR, as has already been reported in other global studies (p=0.002) (A). While HDL levels are a possible risk factor for DR (p<0.001) (B). While LDL levels as reported above did not present a factor to develop DR (p=0.83), globally (C). Maximum Likelihood Model (MLE).

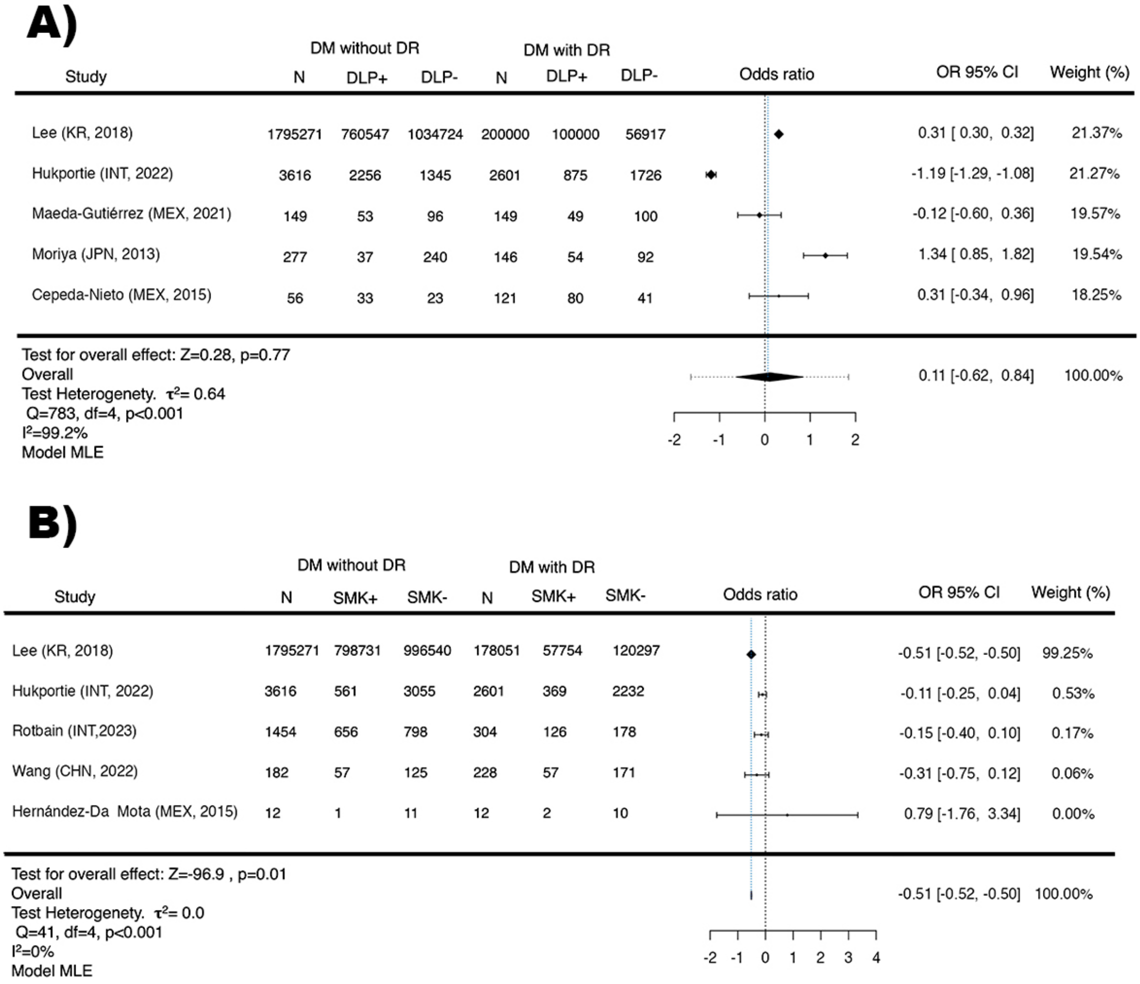

Supplementary Figure S3. DLP and SMK forest plots with and without RD. The diagnosis of dyslipidemia is not a risk factor for DR, which is contradictory, as has already been reported in other studies globally ($p = 0.77$) (A). While smoking is a possible risk factor globally for DR ($p<0.01$) (B). Maximum Likelihood Model (MLE).
